# Premature Deaths

**Published:** 1863-02

**Authors:** 


### HALL'S JOURNAL OF HEALTH.

Our Legitimate Scope is almost boundless: for whatever begets pleasurable and harmless feelings, promotes Health; and whatever induces disagreeable sensations, engenders Disease.

WE AIM TO SHOW HOW DISEASE MAY BE AVOIDED, AND THAT IT IS BEST, WHEN SICKNESS COMES, TO TAKE NO MEDICINE WITHOUT CONSULTING A PHYSICIAN.

Vol. X.] FEBRUARY, 1863. [No. 1. 2

### PREMATURE DEATHS.

If the first seven articles which follow are maturely considered, it will be found that very many premature deaths, whether by suicide or other forms of violence, or by disease, are traceable to moral causes; hence it is as much our duty to avoid these as it is to avoid and guard against the physical causes of disease and death. The suicides in France now average ten a day; the number for the present century, thus far, is over three hundred thousand. Not a day passes in which a suicide may not be directly traced to want of success in life; to the false moralities inculcated by wicked or ignorant writers; to the failure of parents in obtaining a proper influence over their children; to unrestrained appetites and passions; and to the inability of multitudes "to get along in the world" prosperously, for want of thoroughness of preparation for their calling or station in life.

---

